# Transarterial chemoembolization combined with radiofrequency ablation for medium and large hepatocellular carcinoma: insufficient ablation is associated with intrahepatic distant metastasis and extrahepatic metastasis

**DOI:** 10.3389/fonc.2024.1283843

**Published:** 2024-04-05

**Authors:** Peng Guo, Junjun Zheng, Xingtao Pi, Feng Gao, Yushan Zhao, Chunming Xie, Wendong Cao

**Affiliations:** ^1^ Shanxi Bethune Hospital, Shanxi Academy of Medical Sciences, Tongji Shanxi Hospital, Third Hospital of Shanxi Medical University, Taiyuan, China; ^2^ Tongji Hospital, Tongji Medical College, Huazhong University of Science and Technology, Wuhan, China; ^3^ The Third People’s Hospital of Datong, Datong, China; ^4^ Shanxi Provincial People’s Hospital, Taiyuan, China; ^5^ Shanxi Cancer Hospital, Taiyuan, China

**Keywords:** transarterial chemoembolization, radiofrequency ablation, hepatocellular carcinoma, complete ablation, insufficient ablation, metastasis

## Abstract

**Purpose:**

To compare the prognosis of complete and insufficient ablation of transarterial chemoembolization (TACE) combined with radiofrequency ablation (RFA) in treating medium and large hepatocellular carcinoma (HCC) and to explore the differences in recurrence patterns between the two groups

**Patients and methods:**

Patients´ medical records and imaging data of patients with confirmed HCC from January 2014 to January 2022 were collected. These patients were divided into 2 groups: complete ablation (n=172) and insufficient ablation (n=171). Overall survival (OS) and progression-free survival (PFS) were estimated by the Kaplan-Meier curve and the log-rank test was used to compared. Fisher’s exact test was used to compare recurrence patterns between the two groups.

**Results:**

The median OS time was 72.8 months (95%CI:69.5-76.1) and 62.0 months (95%CI: 55.3-68.7) in the complete and insufficient ablation groups, respectively. The median PFS time in the complete ablation group was 67.8 months (95% CI: 65.2-70.4) and 38.6 months (95%CI: 29.8-47.4) in the insufficient ablation group. The OS and PFS rates of the complete ablation group were significantly better than those of the insufficient ablation group (*P*<0.001). In the complete ablation group, 25(41%) patients experienced local tumor progression(LTP), 36(59%) experienced intrahepatic distant progression(IDP), and 0(0%) experienced extrahepatic progression (EP). In the insufficient ablation group, 51 (32.1%) patients experienced LTP, 96 (60.4%) experienced IDP, and 12 (7.5%) experienced EP. The progression patterns of the two groups were statistically significant (*P*=0.039).

**Conclusion:**

Insufficient ablation indicates a poor survival outcome of TACE combined with RFA for medium and large HCC and can promote intrahepatic distant and extrahepatic metastasis.

## Introduction

Hepatocellular carcinoma accounts for around 90% of liver cancers, has become a major health issue (1). Various treatment methods are available for HCC, including liver resection, ablation (such as radiofrequency and microwave ablation), liver transplantation, TACE, hepatic arterial infusion chemotherapy(HAIC), and systemic therapies like sorafenib, lenvatinib, PD1 or PD-L1 (2, 3). Radiofrequency ablation is the most commonly used image-guided ablation technique and has been found to be as effective as surgery resection for early-stage hepatocellular carcinoma (4). For middle-stage HCC, TACE is the most commonly used treatment (5).

TACE combined with RFA has been shown to achieve better results than either treatment alone (6–8), particularly for tumors less than 7cm in diameter (9). For larger tumors, TACE can be used to downstage the tumor before performing RFA. Prospective randomized trials have shown that TACE combined with RFA can achieve satisfactory results for HCC (10–12).

Complete ablation and insufficient ablation are both considered as independent risk factors that impact the prognosis of radiofrequency ablation(RFA) in treating hepatocellular carcinoma (HCC) (13–15). We hypothesize that these two factors also play a significant role in transarterial chemoembolization(TACE) combined with RFA, and that the progression patterns may differ between the two groups. However, there is a lack of research comparing the prognosis and progression patterns of complete ablation and insufficient ablation in TACE combined with RFA. Through this retrospective study, we aim to compare the prognosis and progression patterns in medium and large HCC patients who undergo TACE combined with RFA, with a particular focus on complete and insufficient ablation.

## Patients and methods

### Patients

The patients´ medical records and imaging data were analyzed at Shanxi Bethune Hospital and Shanxi Provincial People’s Hospital between January 2014 and January 2021. All patients were diagnosed with HCC and underwent combined TACE and RFA treatment. The diagnosis of hepatocellular carcinoma(HCC) was determined using non-invasive criteria or biopsy. The non-invasive diagnostic criteria for HCC were based on the guidelines provided by the European Association for the Study of the Liver. These criteria involved the presence of liver cirrhosis, a tumor diameter larger than 1 cm, and arterial hypervascularization with venous or delayed phase washout observed through multi-detector computed tomography (MDCT) or dynamic magnetic resonance imaging(MRI) (1, 16).

Inclusion criteria include: The tumor diameter is greater than 3cm or the tumor diameter is less than 3 layers but the number of tumors is greater than 3. Participants did not have vascular invasion or extrahepatic metastasis, had an ECOG score of 0 or 1, and were classified as Child-Pugh class A or B. Exclusion criteria include patients with a history of liver transplantation, ablation, or surgical resection, loss to follow-up, or concomitant tumors in other locations.

Given that the study was conducted retrospectively and anonymously, the requirement for informed consent was waived. This study has obtained ethical approval by Shanxi Bethune Hospital (2020-109) and Shanxi provincial people´s Hospital.

### TACE-procedure

The TACE procedure was carried out by clinicians with over 8 years of experience in TACE. Under the guidance of digital subtraction angiography, the procedure was performed under local anesthesia. In order to identify all arteries that supplied the tumors, angiography of the superior mesenteric artery, phrenic artery, and internal thoracic artery was conducted as deemed necessary using a 5F catheter (RH catheter; Cook, Bloomington). After identifying all the supplying arteries, the catheter was selectively advanced to the specific artery supplying the tumors using 3F microcatheters (Progreat; Terumo, Japan). In order to minimize liver function damage, the microcatheter was guided to the lesion vessels, such as the subsegmental vessels. To embolize the tumor-feeding arteries, a mixture of iodized oil (Lipiodol; Guerbet, France) and epirubicin (50 mg/m^2^) was employed, followed by polyvinyl alcohol particle embolization. After embolization, angiography was performed to confirm complete occlusion of the feeding artery. The endpoint of TACE is the absence of tumor vascular opacification on post-embolization angiography.

### RFA-procedure

RFA is typically conducted one month after TACE, unless the tumor’s diameter exceeds 7 cm or there are more than 3 tumors. In such cases, TACE is used to downstage the tumor until the diameter is less than 7 cm or the number of tumors is less than 3 before RFA is performed. The RFA procedure is carried out by experienced clinicians, guided by CT or ultrasound, and performed percutaneously under local anesthesia. The radiofrequency generator system (S-1500, MedSphere International Inc., CA, USA) is used, with a monopolar electrode for tumors smaller than 2cm and an expandable multi-tined electrode (alone or in combination with a monopolar electrode) for larger tumors. The power and duration of the procedure are determined by the manufacturer’s recommendations and the size of the lesions, with expanding or overlapping ablation performed to ensure complete ablation.

### Determination and assessment

Complete ablation or insufficient ablation was determined by two diagnostic radiologists with over 5 years of experience based on enhanced CT or MR imaging after the radiofrequency ablation(RFA) procedure. Complete ablation is defined as the ablative area exceeding the lesion by ≥5mm, otherwise, it is defined as insufficient ablation (8, 9). This study utilized a specific software to analyze the ablation margin. Imaging fusion was accomplished by utilizing cutting-edge commercial software for rigid imaging registration. Initially, automatic registration was employed and the results were subsequently verified. The arterial phase was specifically chosen as the optimal image for pre-ablation assessment due to its ability to clearly distinguish the lesions from the surrounding liver tissue, facilitating an accurate evaluation of the ablation margin. Conversely, the venous phase was utilized as the dataset for post-ablation images. Post the integration of pre- and post-ablation images, the separations between the tumor boundary and the edge of the necrosis zone in axial, coronal, and sagittal planes were meticulously gauged. The most minimal among these distances was established as the ablation margin. Overall survival(OS) was determined as the duration from the initial radiofrequency ablation(RFA) procedure until the occurrence of death or the last follow-up. Recurrence-free survival(RFS) was defined as the duration from the first RFA procedure until the occurrence of tumor recurrence or the last follow-up. Tumor recurrence encompassed local tumor progression(LTP), intrahepatic distant recurrence(IDR), and extrahepatic recurrence(ER) (17). LTP was characterized as the emergence of a lesion either within the ablation area or at the periphery of the ablation zones, as determined by enhanced CT or MR imaging following radiofrequency ablation(RFA) (18, 19). Intrahepatic distant recurrence (IDR) is defined as the occurrence of new tumors in distinct subsegments of the liver subsequent to radiofrequency ablation (RFA), or the emergence of new tumors within the same liver subsegment that are not adjacent to the ablation zone, as identified by enhanced CT or MR imaging (20, 21). ER was characterized as the emergence of new metastatic lesions outside of the liver subsequent to radiofrequency ablation (21). Early recurrence is defined as the reappearance of tumors within a period of two years following the radiofrequency ablation(RFA) procedure (22). On the other hand, late recurrence is characterized as the recurrence of tumors that manifest two years or more after the RFA procedure has been conducted (17).

If the diameter of the tumor is greater than 7 cm or the number is more than 3, downstaging by TACE was performed first. Successful downstaging was defined as reduction in tumor size(≤7cm) and tumor number(≤3) by enhanced CT or MR imaging. When assess the downstaging, the whole tumor was treated not only the contrast-enhanced part of the tumor (10).

### Follow -up protocol

The first follow-up visits were conducted one month after the RFA procedure. If no issues were identified during the initial follow-up, patients were monitored every two months in the first year, followed by every three months for the following three years. The study’s final follow-up date was September 1, 2021. Follow-up assessments included abdominal CT or MRI with contrast, chest CT scans, and blood tests measuring platelet (PLT) count, bilirubin, aspartate aminotransferase (AST), alanine aminotransferase (ALT), albumin (ALB), prothrombin time (PT), and serum α-fetoprotein (AFP) levels.

If a recurrence was detected, patients were treated with RFA, transarterial chemoembolization, sorafenib, or lenvatinib based on the tumor’s location, liver function, and the patient’s overall condition.

### Statistical analysis

Comparisons between the complete ablation and insufficient ablation groups were done using the Pearson χ^2^ and fisher exact tests test for categorical data. Univariate and multivariate analyses were conducted using Cox regression to assess the impact of various factors on overall survival (OS) and recurrence-free survival(RFS). If the result of the univariate analysis is *P*<0.05, multivariate analysis will be conducted. The Kaplan-Meier curve was used to estimate OS and RFS, and a log-rank test was employed to compare the survival outcomes between two groups. Patients who were still alive at the last follow-up examination were considered as censored data. All statistical tests were two-tailed, and a P-value of less than 0.05 was considered statistically significant. The statistical analyses were performed using SPSS statistical software (version 23.0, Chicago).

## Results

### Study population

**Figure 1:**
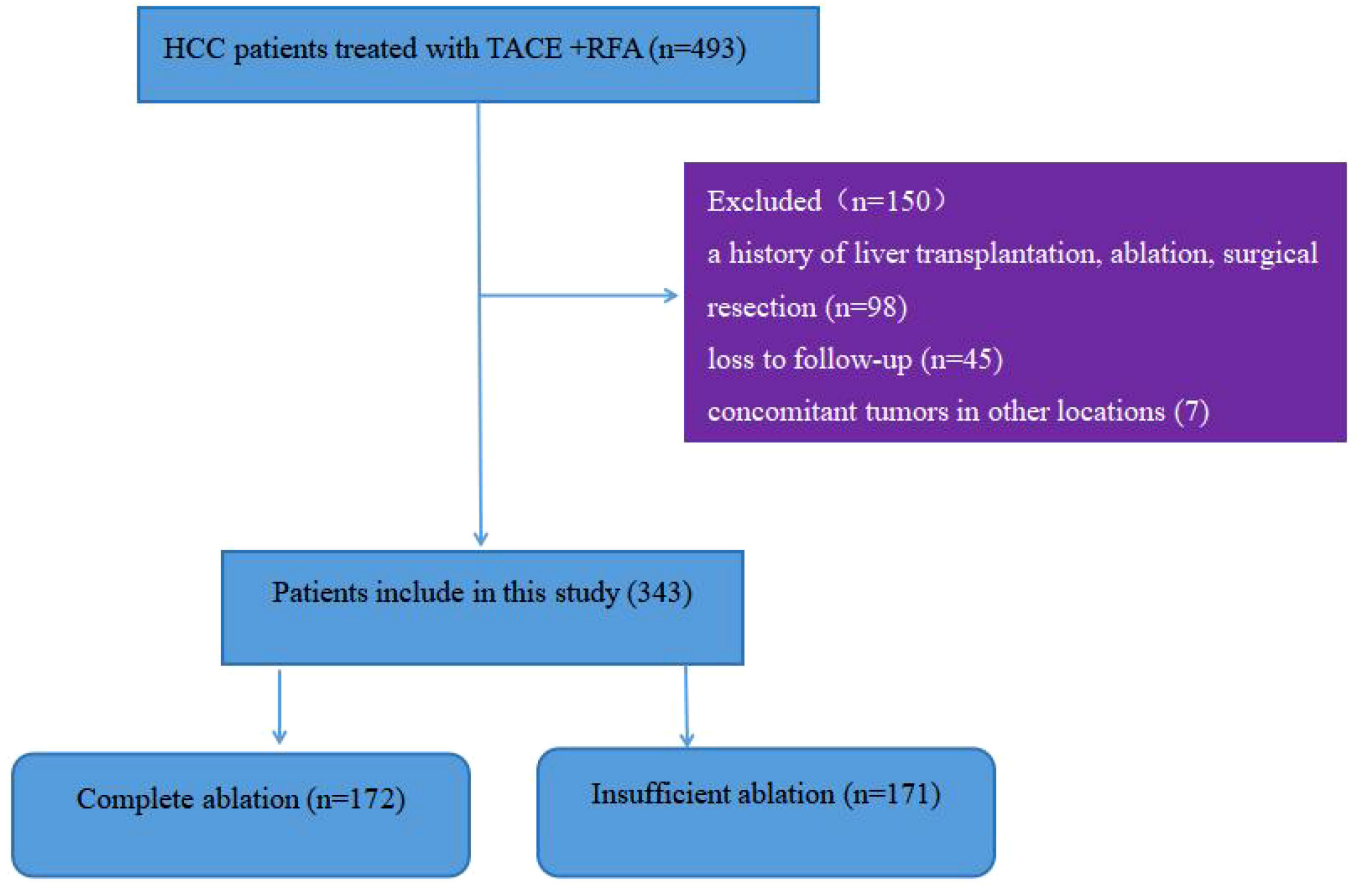
Flowchart for the patient selection.

During the study period, a total of 493 patients with hepatocellular carcinoma underwent TACE combined with RFA treatment. **Figure 1** shows that 150 patients were excluded because they did not meet the inclusion criteria, leaving 343 patients for analysis, with 172 in the complete ablation group and 171 in the insufficient ablation group. **Table 1** compares the baseline characteristics (such as age, gender, maximum tumor diameter, tumor number, HBV infection ratio, AFP level, PLT level, AST level, ALBI score, Child-Pugh class, BCLC period, ECOG score, and treatment with or without sorafenib or lenvatinib) of the two groups. The ALT level was significantly higher in the insufficient ablation group than in the complete ablation group (*P=*0.010), while no significant differences were observed in other characteristics between the two groups.

**Table 1:** Baseline characteristics of HCC received TACE combined with RFA.

Characteristics		Complete ablation (n=172)	Insufficient ablation (n=171)	P value
Age (years)	≤60	89 (51.7)	103(60.2)	0.128
	> 60	83 (48.3)	68 (39.8)	
Gender	Male	148 (86.0)	153(89.5)	0.411
	Female	24 (14.0)	18 (10.5)	
MTD (cm)	3.1–5	103(59.9)	90(52.6)	0.194
	5.1–7	35(20.3)	33(19.3)	
	>7*	34(19.8)	48(28.1)	
Tumor number	≤3	148(86.0)	136(79.5)	0.118
	> 3*	24(14.0)	35(20.5)	
HBV	Yes	157 (91.3)	156(91.2)	1.000
	No	15 (8.7)	15(8.8)	
AFP (ng/mL)	≤400	118 (68.6)	110 (64.3)	0.425
	> 400	54 (31.4)	61 (35.7)	
PLT	≤100	48(27.9)	44 (25.7)	0.715
	> 100	124 (72.1)	127 (74.3)	
ALT (U/L)	≤40	99 (57.6)	74(43.3)	0.010
	> 40	73 (42.4)	97(56.7)	
ALBI score	1	123 (71.5)	110 (64.3)	0.306
	2	48 (27.9)	60 (35.1)	
	3	1 (0.6)	1 (0.6)	
Child–pugh class	A	161(93.6)	160(93.6)	1.000
	B	11(6.4)	11(6.4)	
BCLC period	A	106(61.6)	92(53.8)	0.156
	B	66 (38.4)	79(46.2)	
ECOG score	0	110(64.0)	124(72.5)	0.104
	1	62 (36.0)	47 (27.5)	
Sorafinib/Lenvatinib	Yes	36(20.9)	47(27.5)	0.167
	No	136(79.1)	124(72.5)	

MTD, maximum tumor diameter; HBV, hepatitis B virus; AFP alpha–fetoprotein; PLT, platelets; ALT alanine aminotransferase; ALBI albumin–bilirubin; BCLC, barcelona clinic liver cancer; ECOG, eastern cooperative oncology group performance status.

*If the diameter of the tumor is greater than 7 cm or the number is more than 3, downstaging by TACE was performed.

### Overall survival

**Figure 2:**
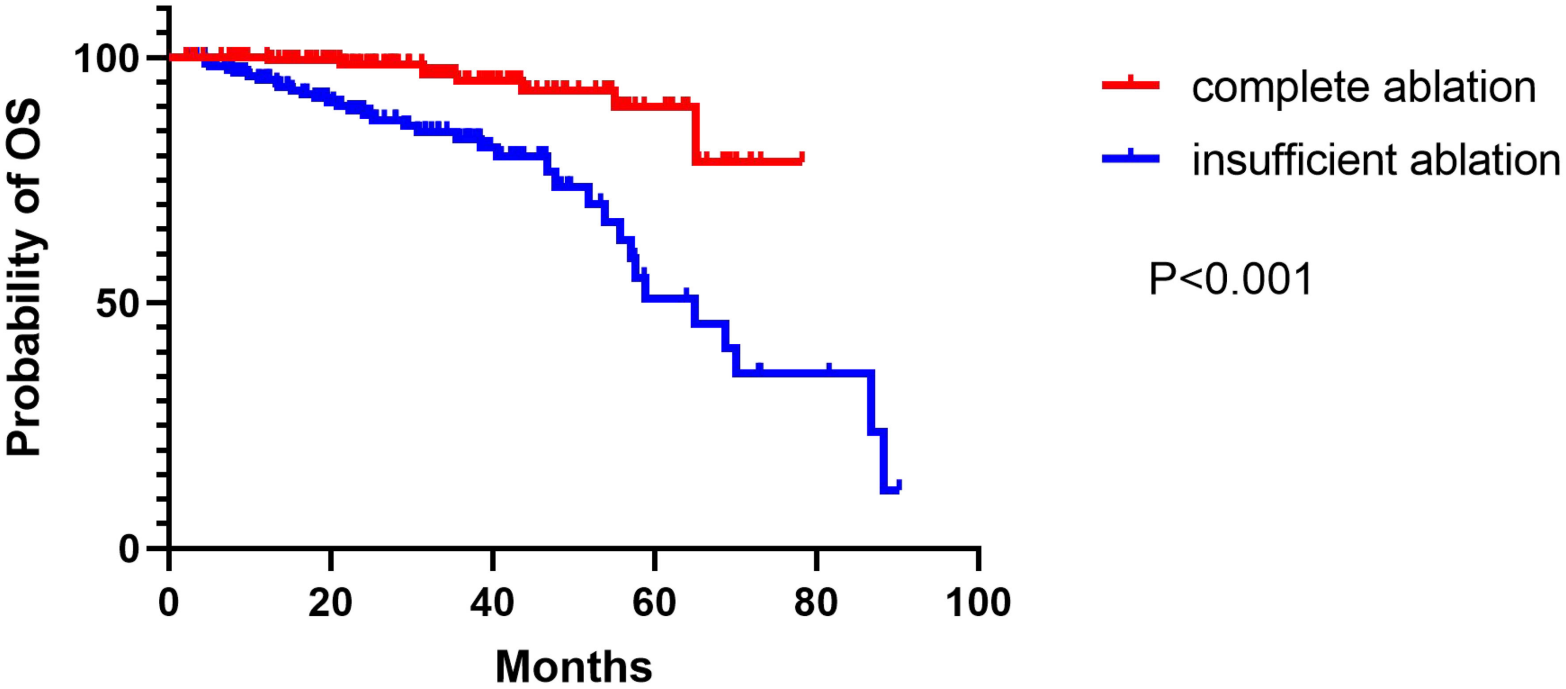
The Kaplan–Meier curve showed the difference of OS rates between the complete ablation and the insufficient ablation group.

The median duration of follow-up was 52.0 months (range: 6–88 months) in the group that underwent complete ablation, and 50.0 months (range: 7–90.2 months) in the group that had insufficient ablation. Throughout the follow-up period, 9 patients from the complete ablation group and 35 patients from the insufficient ablation group died. The median overall survival (OS) time in the complete ablation group was 72.8 months (95% confidence interval [CI: 69.5-76.1]), while in the insufficient ablation group, it was 62.0 months (95%CI:55.3-68.7). The Kaplan-Meier curve demonstrated that the OS rates were significantly higher in the complete ablation group compared to the insufficient ablation group (*P* < 0.001; **Figure 2**). Both univariate and multivariate Cox regression analyses identified complete ablation and the use of sorafenib and lenvatinib therapy as risk factors for OS (**Table 2**).

**Table 2:** Univariate and multivariate analysis of prognostic factors for OS.

Variables (OS)	Univariate Analysis			Multivariate Analysis		
	HR	95% CI	*P–value*	HR	95% CI	*P–value*
Complete ablation						
Yes	0.207	0.099–0.433	<0.001	0.199	0.095–0.417	<0.001
No						
Age (years)						
≤60	1.367	0.755–2.475	0.303	NA	NA	NA
>70						
Gender						
Female	0.986	0.411–2.369	0.975	NA	NA	NA
Male						
MTD (cm)						
3.1–5	1.091	0.707–1.684	0.693	NA	NA	NA
5.1–7	1.607	0.962–2.685	0.070	NA	NA	NA
> 7						
Tumor Number						
< 3	0.653	0.232–1.834	0.418	NA	NA	NA
≥3						
HBV						
Yes	1.127	0.431–2.944	0.808	NA	NA	NA
No						
AFP (ng/ml)						
≤400	1.349	0.733–2.480	0.336	NA	NA	NA
>400						
ALT (U/L)						
≤40	1.463	0.806–2.656	0.211	NA	NA	NA
>40						
PLT (U/L)						
≤100	0.888	0.461–1.712	0.723	NA	NA	NA
>100						
ALBI score						
1	1.124	0.614–2.056	0.704	NA	NA	NA
2						
3						
Child–pugh class						
A	0.045	0.000–23.986	0.333	NA	NA	NA
B						
BCLC period						
A	0.746	0.405–1.374	0.347	NA	NA	NA
B						
ECOG score						
0	0.809	0.443–1.477	0.491	NA	NA	NA
1						
Sorafenib/Lenvatinib						
Yes	0.261	0.093–0.731	0.011	0.243	0.086–0.681	0.007
No						

OS, overall survival; NA, not applicable; MTD, maximum tumor diameter; HBV, hepatitis B virus; AFP, alpha–fetoprotein; PLT, platelets; ALT, alanine aminotransferase; ALBI, albumin–bilirubin; BCLC, barcelona clinic liver cancer; ECOG, eastern cooperative oncology group performance status.

### Recurrence-free survival

#### Recurrent patterns of two groups

**Figure 3:**
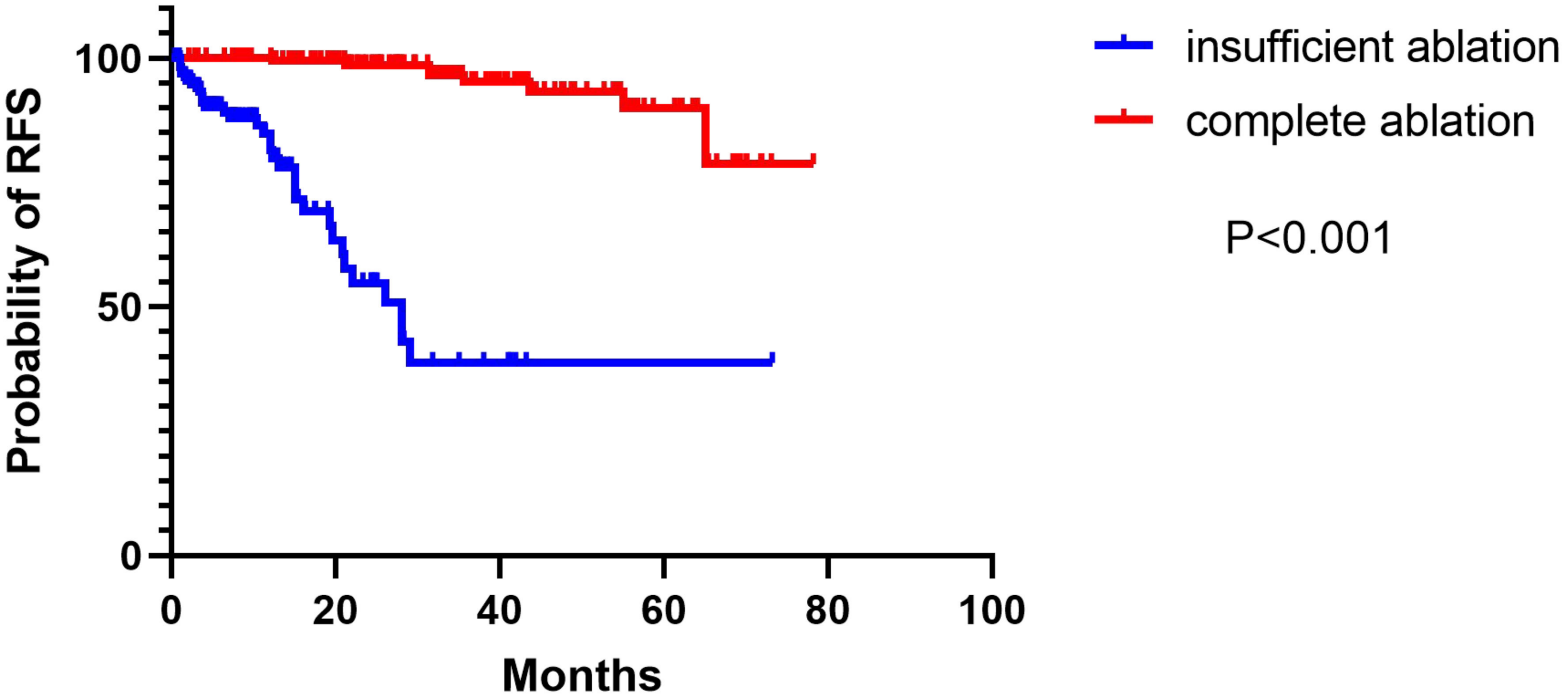
The Kaplan–Meier curve showed the difference of RFS rates between the complete ablation and the insufficient ablation group.

During the follow-up period, 61 patients relapsed in the complete ablation group and 159 patients relapsed in the insufficient ablation group. The median RFS time was 67.8 months (95% CI: 65.2-70.4) in the complete ablation group and 38.6 months (95%CI: 29.8-47.4) in the insufficient ablation group. The Kaplan-Meier curve indicated a significantly better RFS rate in the complete ablation group compared to the insufficient ablation group (*P*<0.001; **Figure 3**). According to univariate and multivariate Cox regression analyses, complete ablation was identified as a risk factor for RFS (**Table 3**). Among the 61 patients who relapsed in the complete ablation group, 36 received RFA, 25 received TACE treatment, and 36 of these patients were treated with sorafenib or lenvatinib. In the insufficient ablation group, 98 patients received RFA and 61 patients received TACE treatment, with 47 of these patients receiving sorafenib or lenvatinib.

**Table 3:** Univariate and multivariate analysis of prognostic factors for RFS.

Variables (RFS)	Univariate Analysis			Multivariate Analysis		
	HR	95% CI	*P–value*	HR	95% CI	*P–value*
Complete ablation						
Yes	0.207	0.099–0.433	<0.001	0.102	0.048–0.216	<0.001
No						
Age (years)						
≤60	0.939	0.397–2.223	0.385	NA	NA	NA
>70						
Gender						
Female	1.300	0.719–2.350	0.886	NA	NA	NA
Male						
MTD (cm)						
3.1–5	1.260	0.819–1.937	0.293	NA	NA	NA
5.1–7	1.388	0.837–2.303	0.204	NA	NA	NA
> 7						
Tumor Number						
< 3	0.543	0.194–1.517	0.244	NA	NA	NA
≥3						
HBV						
Yes	0.984	0.388–2.498	0.973	NA	NA	NA
No						
AFP (ng/ml)						
≤400	1.466	0.798–2.693	0.217	NA	NA	NA
> 400						
ALT (U/L)						
≤40	1.439	0.794–2.606	0.230	NA	NA	NA
> 40						
PLT (U/L)						
≤100	0.951	0.497–1.817	0.818	NA	NA	NA
> 100						
ALBI score						
1	1.096	0.594–2.026	0.769	NA	NA	NA
2						
3						
Child–pugh class						
A	0.045	0.000–12.604	0.281	NA	NA	NA
B						
BCLC period						
A	0.898	0.489–1.648	0.728	NA	NA	NA
B						
ECOG score						
0	0.712	0.393–1.291	0.264	NA	NA	NA
1						

RFS, recurrence–free survival; NA, not applicable; MTD, maximum tumor diameter; HBV, hepatitis B virus; AFP, alpha–fetoprotein; PLT, platelets; ALT, alanine aminotransferase; ALBI, albumin–bilirubin; BCLC, barcelona clinic liver cancer; ECOG, eastern cooperative oncology group performance status.

**Figure 4:**
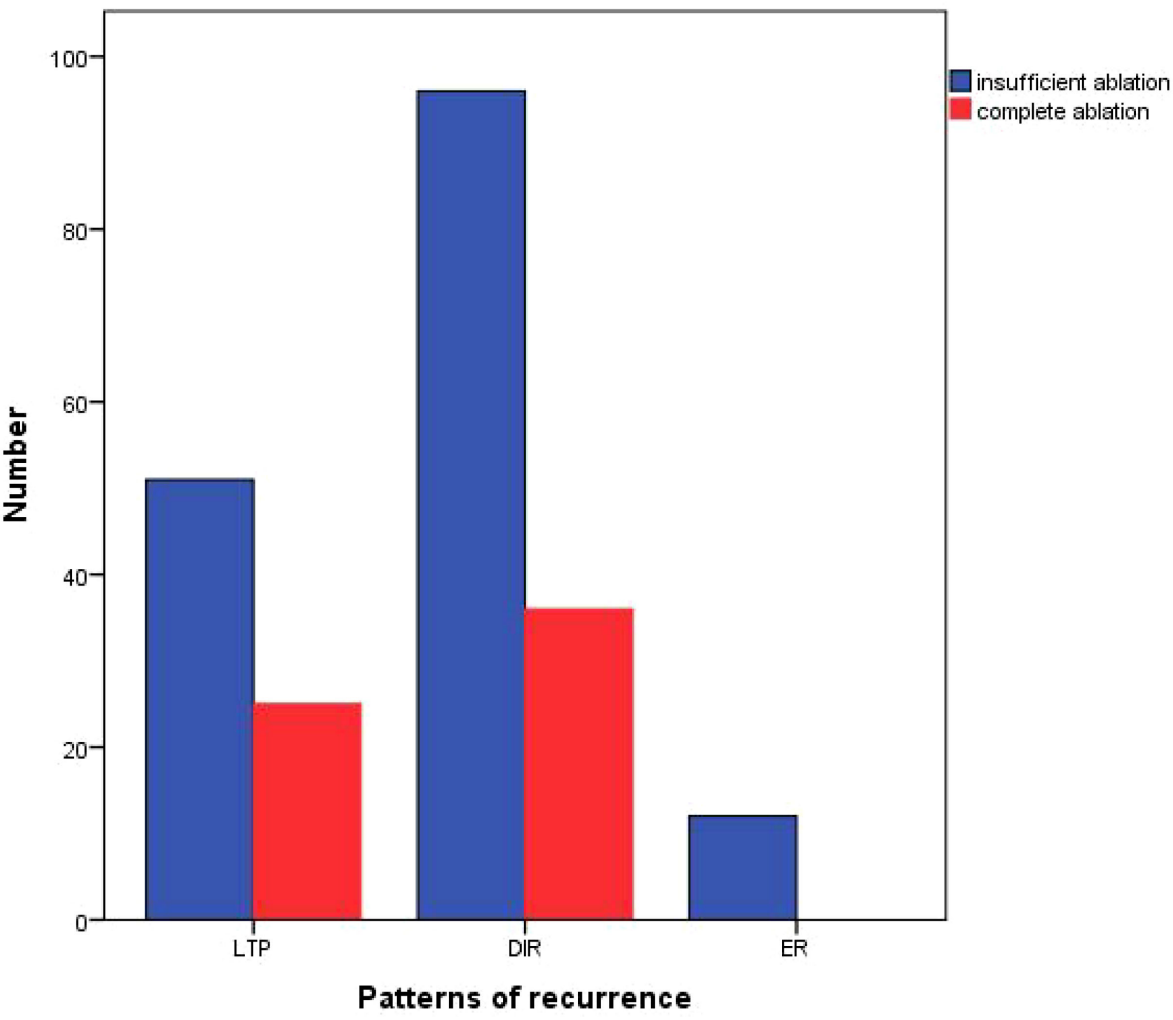
Recurrence patterns of complete ablation and insufficient ablation.

**Figure 5:**
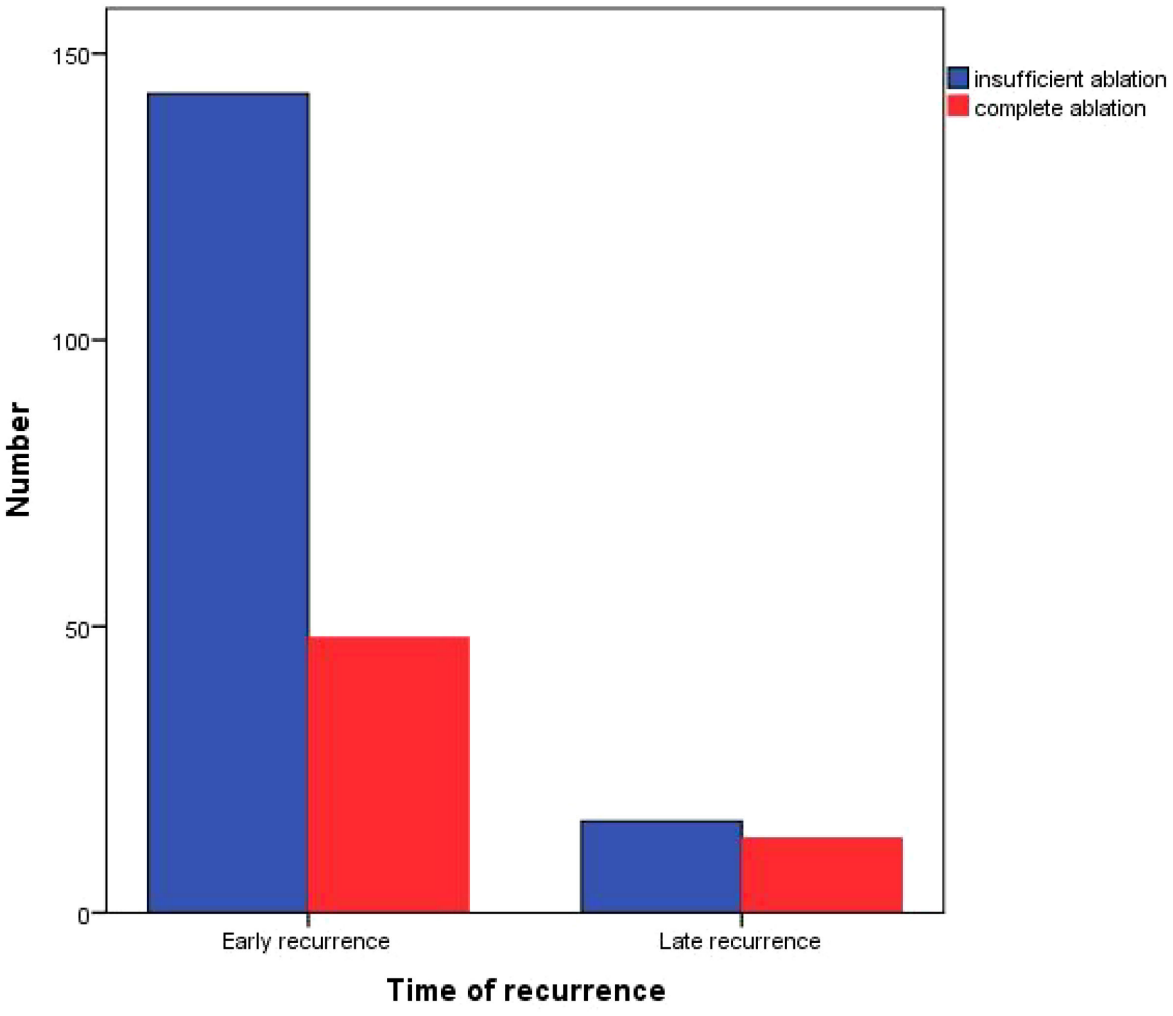
Recurrence time of complete ablation and insufficient ablation.

Among the relapsed patients in the complete ablation group, 25 (41%) patients experienced local tumor progression (LTP), 36 (59%) patients had distant tumor recurrence (DIR), and none (0%) of the cases were early recurrence (ER). In the insufficient ablation group, 51 (32.1%) patients experienced LTP, 96 (60.4%) patients had DIR, and 12 (7.5%) cases were ER (**Figure 4**). The recurrence patterns of the two groups were compared, and the difference was statistically significant (*P*=0.039; **Table 4**). In the complete ablation group, there were 48 (78.7%) cases of early recurrence and 13 (21.3%) cases of late recurrence. In the insufficient ablation group, there were 143 (89.9%) cases of early recurrence and 16 (10.1%) cases of late recurrence (**Figure 5**). The difference in recurrence time was statistically significant between the two groups (*P*=0.046; **Table 4**).

**Table 4:** Recurrent patients of complete ablation and insufficient ablation.

	Complete ablation (n=61)	Insufficient ablation (n=159)	P value
Patterns of recurrence			0.039
LTP	25 (41.0%)	51(32.1%)	
DIR	36 (59.0%)	96 (60.4%)	
ER	0 (0%)	12 (7.5%)	
Recurrence time			0.043
Early recurrence	48 (78.7%)	143 (89.9%)	
Late recurrence	13 (21.3%)	16 (10.1%)	
Time to recurrence			0.006
Medium(months)	48.2	39.3	
95% CI	39.9–56.6	29.8–48.8	
Overall survival			
Medium(months)	69.4	63.2	0.036
95% CI	63.7–75.1	56.3–70.0	

**Figure 6:**
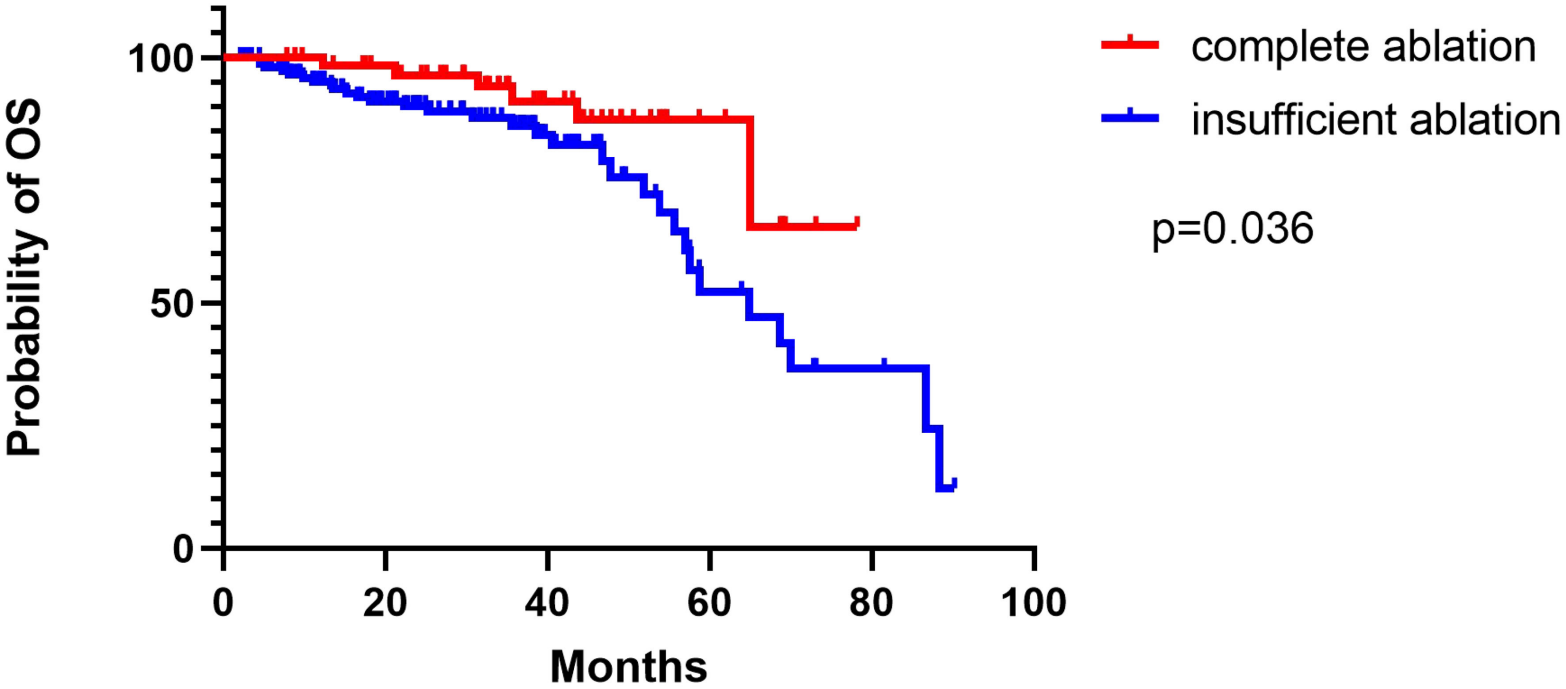
The Kaplan–Meier curve showed the difference of OS rates for relapsed patients.

The median recurrence time of relapsed patients in the complete ablation group was 48.2 months (95% CI: 39.9-56.6). The median recurrence time of relapsed patients in the insufficient ablation group was 39.3 months (95% CI: 29.8-48.8). The Kaplan-Meier curve showed that the recurrence time of the two groups was significantly different (*P*=0.006). The Kaplan-Meier curve also showed that the overall survival rates of relapsed patients in the complete ablation group were significantly better than those in the insufficient ablation group (*P*=0.036; **Figure 6**).

### Complications

There were no treatment-related deaths in our study. The common complications were pain, fever, vomiting, ascites, pleural effusion, bile lakes and hepatic artery portal fistula. During the study, there was no patient occurred more than grade 3 complication.

## Discussion

For patients with hepatocellular carcinoma (HCC) and a tumor diameter of less than 3 cm, radiofrequency ablation (RFA) alone has shown to be effective (23, 24). However, for those with a larger tumor diameter, typically greater than 3 cm, a combination of transarterial chemoembolization and RFA is often used (25–27). This approach has demonstrated good curative effects and safety. The combination of TACE and RFA offers a multitude of advantages. Firstly, TACE not only blocks the flow of blood through the hepatic artery via embolization but also delivers chemotherapeutic agents directly to the tumor site (7). This dual action ensures efficient tumor targeting and enhanced treatment efficacy. Furthermore, the use of iodized oil during TACE serves another crucial purpose. It not only fills the portal vein around the tumor, promoting the accumulation of chemotherapeutic agents, but also aids in the visualization of the tumor during subsequent RFA procedures (28), reducing portal vein flow and ultimately enlarging the area of ablation (29). Despite these benefits, insufficient ablation remains a challenge in the treatment of medium and large HCC with TACE combined with RFA.

In our study, we observed a median overall survival (OS) time of 72.8 months (95% CI: 69.5-76.1) in the complete ablation group, whereas the insufficient ablation group had a median OS time of 62.0 months (95% CI: 55.3-68.7). The Kaplan-Meier curve indicated a significant difference in OS rates between the complete ablation and insufficient ablation groups (*P* < 0.001). Similarly, the complete ablation group showed a median recurrence-free survival (RFS) time of 67.8 months (95% CI: 65.2-70.4), while the insufficient ablation group had a median RFS time of 38.6 months (95% CI: 29.8-47.4). The Kaplan-Meier curve demonstrated significantly better RFS rates in the complete ablation group compared to the insufficient ablation group (*P* < 0.001). Both univariate and multivariate regression analyses confirmed that insufficient ablation independently contributed to an increased risk for both OS and RFS.

We also conducted an analysis of patients with recurrence after complete or insufficient ablation. In the complete ablation group, 25 (41%) patients experienced local tumor progression (LTP), 36 (59%) had distant intrahepatic recurrence (DIR), and 0 (0%) cases had extrahepatic recurrence (ER). In the insufficient ablation group, 51 (32.1%) patients experienced LTP, 96 (60.4%) had DIR, and 12 (7.5%) cases had ER. The incidence of DIR and ER was significantly higher in the insufficient ablation group compared to the complete ablation group (*P*=0.039). Furthermore, there were 48 (78.7%) cases of early recurrence and 13 (21.3%) cases of late recurrence in the complete ablation group, whereas in the insufficient ablation group, there were 143 (89.9%) cases of early recurrence and 16 (10.1%) cases of late recurrence. The incidence of early recurrence was significantly higher in the insufficient ablation group than in the complete ablation group (*P*=0.046). This suggests that insufficient ablation can increase the risk of distant and extrahepatic metastasis. The median recurrence time for relapsed patients in the complete ablation group was 48.2 months, while it was 39.3 months in the insufficient ablation group. The Kaplan-Meier curve showed that the overall survival rates for relapsed patients in the complete ablation group were significantly better than those in the insufficient ablation group (*P*=0.036). This indicates that for two groups of patients with recurrence, the incomplete ablation group also has a worse prognosis.

Insufficient ablation can promote intrahepatic distant metastasis and extrahepatic metastasis through various mechanisms. One mechanism involves sublethal heat treatment, which induces an epithelial-mesenchymal transition (EMT) in hepatocellular carcinoma (HCC) cells. This EMT-like phenotype increases the aggressiveness and growth of HCC cells (30, 31). Additionally, sublethal heat stress triggers a stronger Warburg effect in HCC cells, contributing to their thermotolerance and invasion (32). After insufficient ablation, the stress also causes m6A modification in the EGFR mRNA’s 5’UTR, increasing EGFR and YTHDF1 binding, leading to an increase in EGFR translation and HCC recurrence (33). Insufficient ablation induces autophagy, which promotes residual tumor cell proliferation (34, 35).

This study highlights the significance of adequate ablation in determining the prognosis and recurrence patterns in TACE combined with RFA therapy. Therefore, it is very important to extend the ablation range beyond the lesion by 5mm to achieve complete ablation during the ablation process. In this study, the main reasons for incomplete ablation were the proximity of the lesions to major blood vessels, liver capsule, heart, gallbladder, and bile ducts, among other vital organs. Among the 171 patients in the incomplete ablation group, 51 had lesions close to major blood vessels, 16 had lesions close to the heart, 32 had lesions close to the gallbladder, 37 had lesions close to the bile ducts, and 35 had lesions close to the liver capsule. The proximity to these vital organs may result in incomplete ablation or the inability to ensure an adequate safety margin, ultimately affecting patient survival. Therefore, for lesions located near critical organs, it is recommended to consider TACE treatment first, allowing the tumor to shrink to a size that ensures a sufficient safety margin before proceeding with ablation therapy. Alternatively, a combination of TKIs and immunotherapy can be considered to prevent recurrence.

This study has some limitations. Firstly, being a retrospective study with limited sample sizes, it may suffer from selection bias. Secondly, since most of the cases analyzed were of patients with hepatitis B, the findings may not be generalizable to those with hepatitis C or alcoholic hepatitis. Finally, the study did not differentiate between patients who underwent RFA following downstaging and those who received TACE combined with RFA. In future studies, it may be beneficial to analyze these patient groups separately.

## Conclusion

Insufficient ablation is an independent risk factor for both overall survival and recurrence–free survival in TACE combined with RFA treatment for medium and large HCC. It is indicative of a poor survival outcome and may promote distant metastasis in the liver and extrahepatic metastasis. Insufficient ablation has a significant impact on the patient’s prognosis, and even in cases of recurrence, the prognosis of insufficient ablation is inferior to that of complete ablation.

## Data availability statement

The original contributions presented in the study are included in the article/supplementary material. Further inquiries can be directed to the corresponding author.

## Ethics statement

The studies involving humans were approved by the GCP of Shanxi Bethune Hospital. The studies were conducted in accordance with the local legislation and institutional requirements. The ethics committee/institutional review board waived the requirement of written informed consent for participation from the participants or the participants’ legal guardians/next of kin because as the retrospective and anonymous characteristics, the informed consent from each patient was waived.

## Author contributions

PG: Writing – original draft. JZ: Data curation, Validation, Writing – original draft. XP: Data curation, Methodology, Writing – original draft. FG: Data curation, Investigation, Writing – original draft. YZ: Methodology, Supervision, Writing – review & editing. CX: Conceptualization, Writing – review & editing. WC: Writing – review & editing.
